# Small Finger Osteocutaneous Fillet Flap for Reconstruction in Ring Finger Trauma

**DOI:** 10.1097/GOX.0000000000002477

**Published:** 2019-10-29

**Authors:** Brodie Parent, Liliana Camison, Guilherme Barreiro, Alexander Spiess

**Affiliations:** From the Department of Plastic Surgery, University of Pittsburgh, Pittsburgh, Pa.

## Abstract

Finger amputations are common injuries which result in significant long-term morbidity and loss of function. In this report, we describe a creative operative solution for a 21-year-old man who was in a motorcycle crash and sustained severely comminuted open fractures of the left small and ring fingers with severe crush injury and soft tissue avulsion. Of the tissues and bones in the small finger, only the distal half of the proximal phalanx remained intact and was vascularized via the remaining ulnar neurovascular bundle. In the ring finger, the extensor mechanism and ulnar neurovascular bundle were avulsed and the distal half of the proximal phalanx was absent, but the flexor tendons were intact. A small finger ray amputation was performed. Then, using an osteocutaneous fillet flap based on the ulnar neurovascular bundle from the small finger, the bony gap and soft tissue deficits in the ring finger were reconstructed. The ring finger extensor tendon was then reconstructed. Subsequently, the patient had evidence of bony union on follow-up X-rays and he had a sensate filet flap over the ulnar aspect of the ring finger. This case demonstrates the creative use of a “spare-parts” osteocutaneous fillet flap in the reconstruction of a traumatic finger injury. This example highlights the importance of assessing all available reconstructive options to avoid the morbidity of a finger amputation.

It is estimated that >30,000 patients are treated annually in the United States for finger amputations.^1^ These injuries can have devastating functional consequences for patients. Reconstructive surgeons have the technical ability to preserve threatened digits using soft tissue flaps and spare patients the morbidity of a finger amputation. Finger reconstruction after trauma using composite tissue flaps can have good long-term results, with some series reporting 40 degrees motion in the metacarpophalangeal joint and over 70 degrees of motion in the interphalangeal joints.^2^ In this case report, we describe a small finger osteocutaneous fillet flap based on the ulnar digital artery used to reconstruct a bony and soft tissue deficit at the level of the adjacent ring finger proximal phalanx.

## PREOPERATIVE EVALUATION

A 21-year-old helmeted male motorcycle driver was transported to our level-one trauma center after being struck by a car. During his initial workup with the acute care trauma service, the patient was found to be hypotensive with evidence of fluid on focused assessment with sonography, so he was taken for exploratory laparotomy (negative for injury). Due to a grade IIIC Gustillo open comminuted left femur and tibia fractures with transection of the left popliteal artery, he underwent an above-knee amputation. After this, he became hemodynamically normal, and external fixation of a right tibia fracture was performed.

**Fig. 1.**
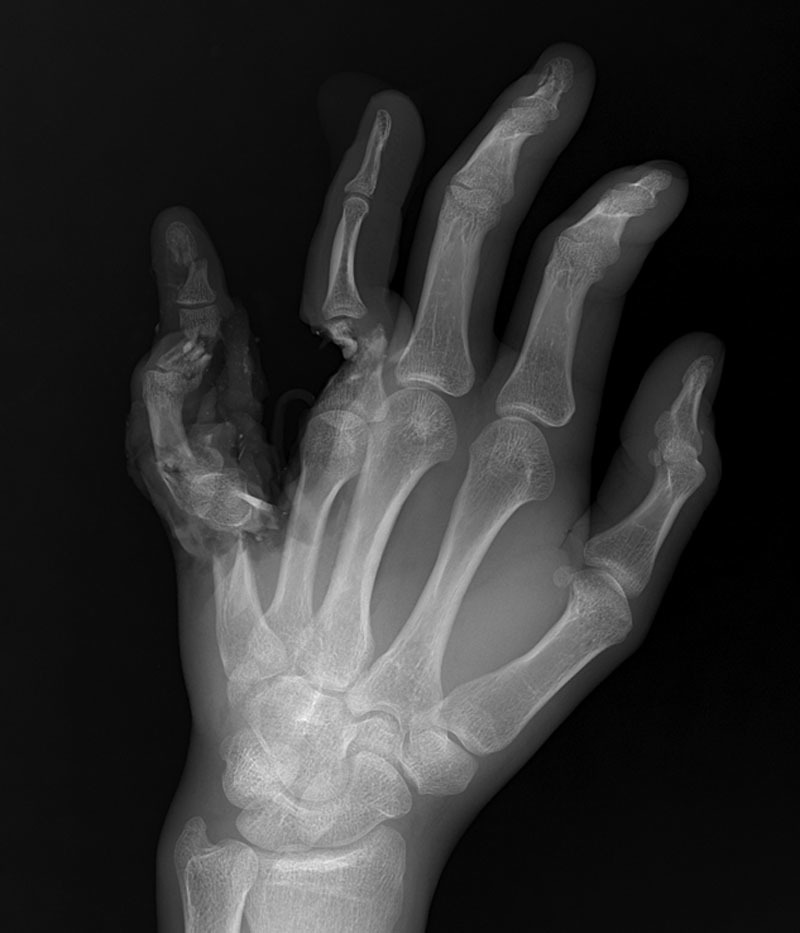
The initial presentation of a left hand injury following a motorcycle crash in a 21-year-old man, showing near complete avulsion and destruction of the small finger, and a bone and soft tissue deficit overlying the ring finger proximal phalanx. Bony injuries included severely comminuted and compound fractures involving the left small finger metacarpal, small finger proximal, middle and distal phalanges, ring finger proximal phalanx, long finger distal phalanx, and distal radius.

At this point, our plastic surgery team was consulted intraoperatively for evaluation and management of severely comminuted and open fractures of the left small and ring fingers with exposed and avulsed tendons and neurovascular bundles. The exam of the small finger demonstrated near complete amputation of soft tissues and bony destruction, except for a 1.5-cm intact skin bridge on the ulnar-volar aspect containing the ulnar neurovascular bundle. The distal half of the proximal phalanx remained attached to this soft tissue flap. Although the small finger was perfused from the remaining ulnar neurovascular bundle, the phalanges and metacarpal had severely comminuted and displaced fractures, with absence of volar cortical bone in the distal metacarpal and the proximal phalanx. The small finger was determined to be non-salvageable due to the severity of these injuries. The exam of the ring finger demonstrated an avulsed extensor tendon and ulnar neurovascular bundle, with soft tissue destruction and bony gap in the distal half of the proximal phalanx. The ring finger flexor tendons and radial neurovascular bundle were intact. Finally, the long finger distal phalanx had a closed comminuted minimally displaced fracture (Fig. 1).

## OPERATIVE COURSE

**Fig. 2.**
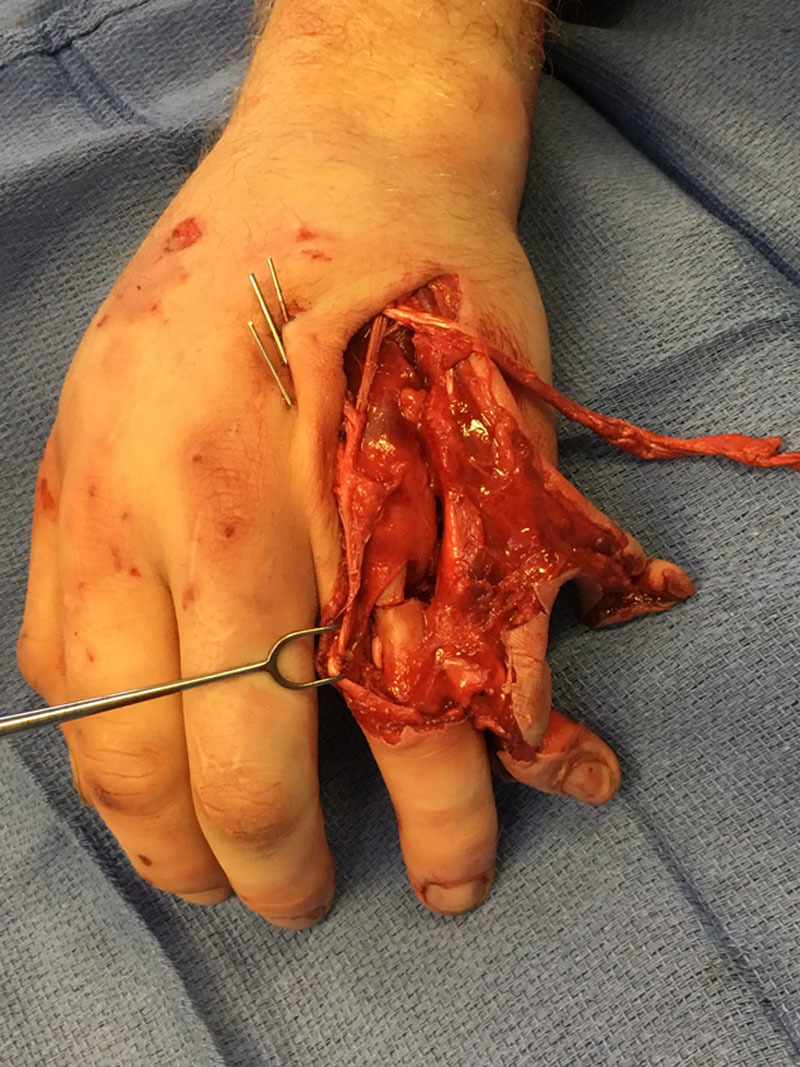
Intraoperative appearance after fixation of the osteocutaneous flap from small finger to ring finger with Kirschner wires.

**Fig. 3.**
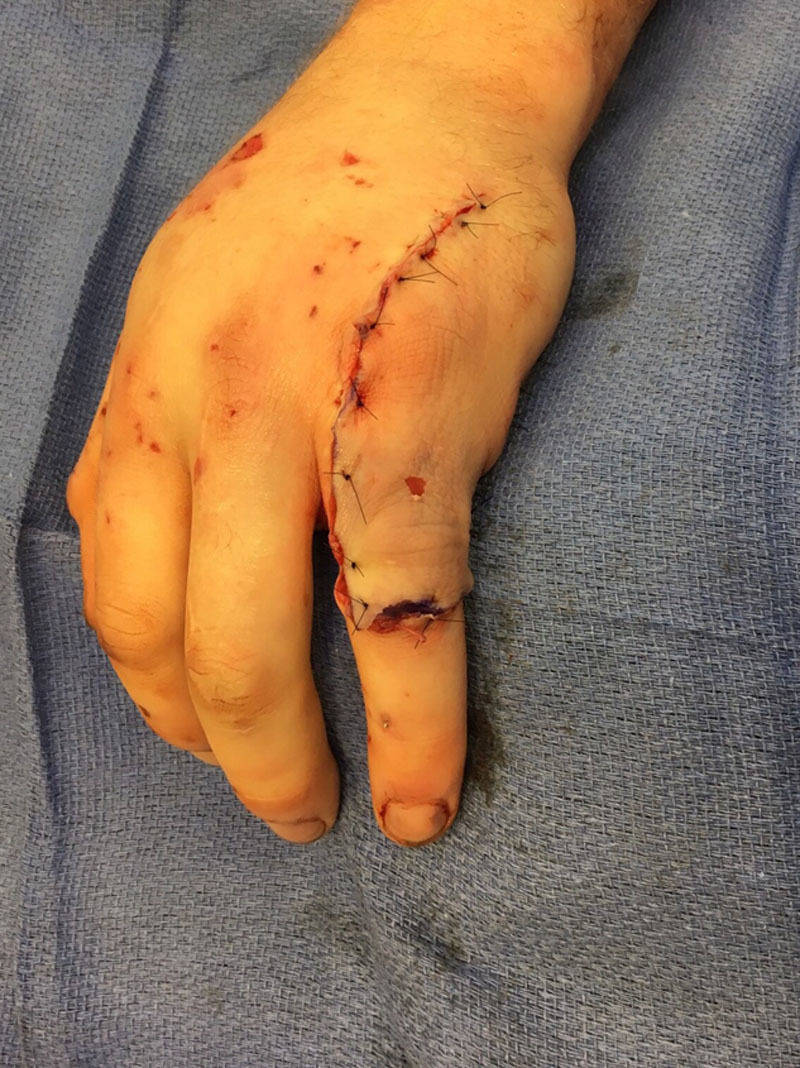
Immediate postoperative appearance after osteocutaneous flap inset from small finger to ring finger.

After determining that the left small finger was non-salvageable, the decision was made to create a pedicled fillet flap with fascia, cutaneous tissue and a vascularized bone graft from the distal half of the proximal phalanx. The small finger ray amputation was performed with a sagittal saw by excising the metacarpal just distal to the carpometacarpal joint. The nonviable soft issues overlying the ring finger were debrided and the finger was irrigated with 3 L of normal saline. The ring finger proximal phalanx fracture site was prepared with a rongeur. Next, a segment of vascularized bone from the small finger proximal phalanx was transferred to the ring finger with fascia and cutaneous tissue from the small finger. This vascularized bone graft was inset using 0.035- and 0.045-inch Kirschner wires. The proximal interphalangeal joint was then reduced using 0.035 Kirschner wires (Fig. 2). The collateral ligaments were repaired. The extensor tendon from the small finger was carried in the fasciocutaneous flap and used to reconstruct the extensor tendon of the ring finger. Bone graft was then packed into the bone defect of the ring finger proximal phalanx. The flap was inset using interrupted 5-0 nylon sutures (Fig. 3). A volar splint was applied.

## POSTOPERATIVE COURSE

**Fig. 4.**
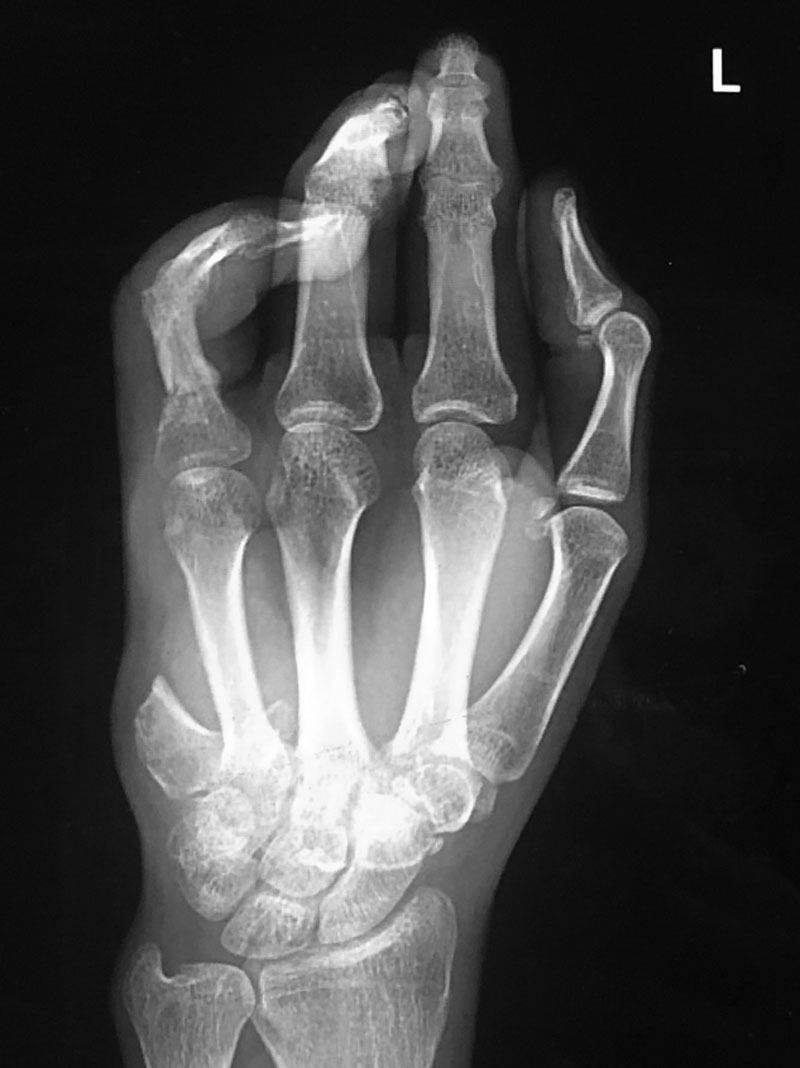
Radiograph demonstrating osteosynthesis at 7-weeks postoperatively after reconstruction of the ring finger using a small finger ulnar digital artery-based osteocutaneous fillet flap.

The patient made an uneventful recovery on the surgical floor following the operation. He reported immediate sensation along the fillet flap on the ulnar side of his ring finger. During the postoperative recovery, the patient was maintained in a volar splint, with active and passive range of motion exercises starting at 1 month. After 6 weeks, the patient underwent manipulation of the metacarphalangeal and interphalangeal joints of the left ring finger, with removal of deep hardware (Fig. 4). At 4 months postoperatively, the patient demonstrated ~30 degrees and 20 degrees of passive motion at the level of the proximal interphalangeal joint and metacarpophalangeal joint, respectively.

## DISCUSSION

The young man described in this case report is a mechanic who depends on the function of his hands and fingers for his livelihood. Amputations are commonly performed for crush injuries resulting in composite soft tissue and bone loss to the digits,^1^ but reconstruction may be indicated depending on the patient’s age, occupation, level of injury, and hand dominance. Recent series indicate that soft tissue reconstruction can produce quite functional fingers with good quality of life.^2^

Other authors have previously reported osteocutaneous flap reconstruction in hand trauma, including osteocuteaneous radial forearm flaps for thumb defects,^3^ reverse dorsal metacarpal osteocutanesous flaps for phalanx deficits,^4^ and even osteocutaneous groin flaps using iliac crest for large soft tissue and metacarpal defects.^5^ Our approach, using a “spare-parts” osteocutaneous fillet flap for ring finger reconstruction, has not been previously reported in the literature to our knowledge. The most recent analogous approach is described by a group of authors in Greece in 2009.^6^ Their report describes using an osteocutaneous flap from the second metacarpal for an amputated thumb reconstruction.

This report highlights the importance of considering all reconstructive options in the mangled hand, including salvage and harvest of “spare-parts” for reconstruction of remaining digits.
